# The dynamics of prion spreading is governed by the interplay between the non-linearities of tissue response and replication kinetics

**DOI:** 10.1016/j.isci.2024.111381

**Published:** 2024-11-13

**Authors:** Basile Fornara, Angélique Igel, Vincent Béringue, Davy Martin, Pierre Sibille, Laurent Pujo-Menjouet, Human Rezaei

**Affiliations:** 1 Université Paris-Saclay, INRAe, UVSQ, VIM, 78350 Jouy-en-Josas, France; 2 Université Claude Bernard Lyon 1, ICJ UMR5208, CNRS, Ecole Centrale de Lyon, INSA Lyon, Université Jean Monnet, Inria Dracula, 69622 Villeurbanne, France

**Keywords:** Biochemistry, Neuroscience, Biocomputational method

## Abstract

Prion diseases, or transmissible spongiform encephalopathies (TSEs), are neurodegenerative disorders caused by the accumulation of misfolded conformers (PrP^Sc^) of the cellular prion protein (PrP^C^). During the pathogenesis, the PrP^Sc^ seeds disseminate in the central nervous system and convert PrP^C^ leading to the formation of insoluble assemblies. As for conventional infectious diseases, variations in the clinical manifestation define a specific prion strain which correspond to different PrP^Sc^ structures. In this work, we implemented the recent developments on PrP^Sc^ structural diversity and tissue response to prion replication into a stochastic reaction-diffusion model using an application of the Gillespie algorithm. We showed that this combination of non-linearities can lead prion propagation to behave as a complex system, providing an alternative to the current paradigm to explain strain-specific phenotypes, tissue tropisms, and strain co-propagation while also clarifying the role of the connectome in the neuro-invasion process.

## Highlights

• Prion sustainability is influenced by non-linear kinetics and tissue response
• Explaining strain-specific tissue tropisms in prion disease
• Axons guide long-distance extracellular prion spreading
• Tissue mediates the co-propagation of prion strains

## Graphical abstract

**Figure:**
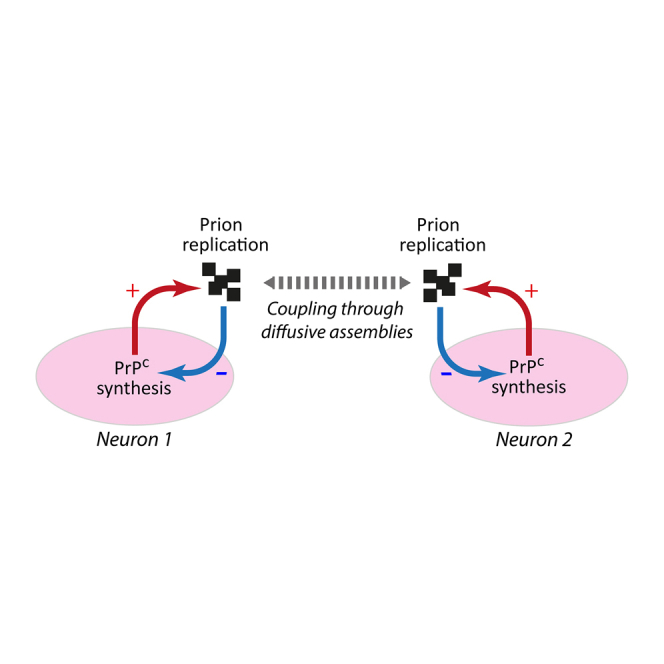

## Introduction

Prion diseases, or transmissible spongiform encephalopathies (TSEs), are a class of untreatable fatal neurodegenerative disorders caused by the accumulation and aggregation in the central nervous system of misfolded conformers (scrapie prion protein: PrP^Sc^) of the host-encoded membrane-anchored prion protein (cellular prion protein: PrP^C^). Throughout the course of the infection, the PrP^Sc^ assemblies replicate by converting PrP^C^ into PrP^Sc^, inducing the extracellular accumulation of the infectious conformers leading to the formation of pathogenic aggregates.^1^^,^^2^ This autocatalytic conversion of PrP^C^ into PrP^Sc^ constitutes the basis of the prion paradigm, which has been extended to Parkinson’s and Alzheimer’s diseases to describe the dissemination process and the progression of these pathologies.^3^

In conventional infectious diseases, variations in clinical manifestations define the pathogenic strain. Similarly, prion diseases are characterized by variations in incubation period, patterns of neuronal loss, PrP^Sc^ deposition, and the biochemical properties of PrP^Sc^ assemblies, which collectively define specific prion strains.^4^^,^^5^ These biochemical properties include the types of fragments generated after proteolysis,^6^^,^^7^ apparent resistance to unfolding,^8^^,^^9^ and the size distribution of PrP^Sc^ assemblies at the terminal stage of the disease.^10^^,^^11^^,^^12^ Recent advancements in prion structural biology have resolved the structure of brain-derived PrP^Sc^ assemblies at atomic resolution for four different prion strains, revealing substantial structural differences.^13^^,^^14^ While variations in the structure of PrP^Sc^ can explain differences in physicochemical properties, templating kinetics, and the dynamics of PrP^Sc^ assemblies,^15^^,^^16^ the link between PrP^Sc^ structure and clinical manifestation remains to be elucidated.

The spatiotemporal progression of tauopathy and misfolded protein deposition in Alzheimer’s and Parkinson’s diseases involves active axonal transport following the neurons axonal connections, the connectome, in a well-established manner called Braak’s directional staging.^17^^,^^18^ In prion diseases, however, the contribution of the connectome to disease progression and histological patterning has yet to be validated.^19^ Indeed, neither the pattern of PrP^Sc^ deposition nor the lesional profile follows the connectome, as they appear to depend solely on the biochemical properties of the strain.^19^ Based on experimental observations, a correlation between levels of PrP^C^ expression and PrP^Sc^ deposition is far from being a general rule.^20^^,^^21^ To explain how strain properties define the lesional profile and PrP^Sc^ deposition, one prevalent but experimentally unsupported hypothesis is the local PrP^C^ conformome hypothesis.^22^ This hypothesis posits that the PrP^C^ expressed by different cell types or brain regions exhibits structural variations, resulting in distinct physicochemical properties.^23^ Consequently, a prion strain would preferentially target specific brain regions based on the compatibility between the strain’s PrP^Sc^ assemblies and the local PrP^C^ physicochemical properties.^22^^,^^23^ However, several experimental observations contradict this theory. Firstly, the absence of PrP^C^ folding intermediates and the highly reversible two-step unfolding/refolding process of native PrP^24^^,^^25^ contradicts the existence of PrP^C^ conformers. Secondly, the high efficiency of conversion in Protein Misfolding Cyclic Amplification (PMCA) conditions, using distinct cell lysates from different brain regions,^26^ invalidates cell-specific templating differences. Thirdly, PrP^Sc^ deposition patterns depend on the inoculation pathway and dose, allowing us to disregard potential impacts from local cofactors.^27^^,^^28^^,^^29^ Fourthly, the spatial and temporal consistency of seeding activity throughout disease progression suggests that each brain area exhibits nearly identical replication efficiency for a specific strain.^30^

Once the local PrP^C^ conformome hypothesis is discarded, another explanation for strain-specific patterns relies on the kinetics underlying replication and dissemination. It has been mathematically and biochemically proven that a reaction-diffusion system, where at least two diffusible reactants interact through non-linear feedback (e.g., catalysis) is capable of self-organizing into defined patterns.^31^^,^^32^ Recent studies on the early stage of prion replication have revealed the formation of two structurally distinct sets of PrP^Sc^ assemblies, chemically tied by a secondary templating pathway.^33^ The study of infectious recombinant prions’ dynamics emphasized catalytic conformational exchanges among various PrP^Sc^ subpopulations.^34^ The dynamics of different PrP^Sc^ subpopulations, alongside local tissue responses affecting PrP^C^ synthesis (such as the Unfolded Protein Response^35^^,^^36^^,^^37^^,^^38^^,^^39^ or variable PrP^C^ production rates^21^), could constitute a new hypothesis. This hypothesis may explain prion strain phenotypes and how two different prion strains, with varying replication rates, could co-propagate within the same individual and evade best replicator selection.^23^^,^^40^^,^^41^^,^^42^ Indeed, co-propagation is often observed in natural prion infections or when adapting a strain to a new host,^42^^,^^43^^,^^44^ where two strains can influence each other. This interaction typically occurs between a fast strain (with a short incubation time) and a slow strain (with a long incubation time). This interference can result in a prolongation of the incubation period or even a blockage of the fast strain.^45^^,^^46^ Although it is widely believed that this interference is primarily driven by hypothetical cofactors or competition between the co-infecting strains for PrP^C^, the exact molecular mechanisms remain unclear.

Therefore, in the present work, we integrated recent advancements in PrP^Sc^ dynamics and tissue responses to prion replication into a reaction-diffusion model, illustrating how prion assemblies disseminate through brain tissue, influenced by local chemical reactions and diffusion processes. To investigate the neuro-invasion, we employed a stochastic approach using the Gillespie algorithm. This method was adapted to simulate both reaction events and diffusion as jump processes across a domain divided into voxels. Our findings demonstrate that the interplay of non-linearities in prion kinetics and tissue response offers a compelling explanation for prion strain phenotypes, co-propagation mechanisms, and tissue tropisms.

## Results

### Interplay between local tissue properties and dynamic nature of PrP^Sc^ assemblies

**Figure 1:**
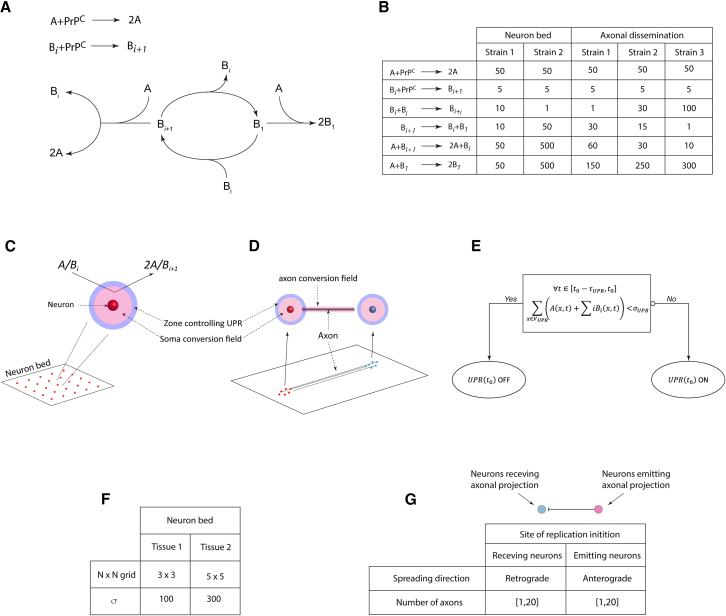
Simulation configurations (A) Kinetic scheme describing prion replication and structural diversification. Experimental observations indicate that the replication process, independently of the strain, generates two conformationally distinct types of PrP^Sc^ assemblies: small oligomeric objects PrP^Sc^A (denoted as *A*) and assemblies capable of condensing PrP^Sc^B_i_^33^ (denoted as *B*_*i*_ where *i* refers to the size of the object). These two subpopulations undergo catalytic exchanges according to the kinetic scheme.^34^ The reaction constants of the kinetic scheme defines a strain. (B) Table summarizing the kinetic parameters of modeled strains used in our simulations. Based on experimental observations,^15^^,^^16^^,^^33^^,^^34^ the templating activity of the A subpopulation is higher than that of the B subpopulation.^33^ (C and D) The evolution of these strains was computed on different types of modeled tissue: on a homogeneous neuron bed (C) and in the axonal dissemination between two clusters (D). In all simulations, we defined two zones surrounding a neuron. The replication field (in pink) limits the zone where templating of *A* and *B*_*i*_ subpopulations can occur. Since PrP^C^ is an extracellularly anchored protein, this replication field is located around the somas and axons. The UPR controlling area (in blue) defines the zone around the soma where if the number of assemblies ($A+\sum iB_{i}$) exceeds a threshold, denoted σ, the UPR activates until the number of assemblies has remained under the threshold for a lag duration τ. While the UPR is activated, no templating reaction can occur in the associated replication area of the neuron. (E) UPR functional diagram illustrating the UPR activation threshold σ and the deactivation lag τ, which may be region-specific. (F) For neuron bed simulations, tissue 1 (T1) and tissue 2 (T2) are defined by the number of neurons in the NxN square grid and their UPR activation threshold (σ). (G) In the axonal dissemination model, two groups of neurons are connected unilaterally by axons. Simulations were seeded near either the receiving or emitting neurons. When replication was initiated near the receiving neurons, the spreading was classified as retrograde; when initiated near the emitting neurons, it was classified as anterograde. To assess the impact of axonal projections on the spreading process, the number of axons was varied from 1 to 20 under both retrograde and anterograde conditions. The results were also compared to those from neuron groups with the same spatial configuration but without axons (see the method details section).

To determine how prion dynamics and local tissue response interact, we computed the evolution of PrP^Sc^ assemblies using the kinetic scheme detailed in the method details section on a uniform neuron grid, where PrP^C^ expression was modulated by the UPR. We analyzed four strain-tissue combinations, defined by two sets of strain parameters and two sets of tissue parameters (Figures 1B and 1F). Strains 1 and 2 share the same templating activities but differ in their assembly dynamics, with Strain 1 favoring larger assemblies. Tissues 1 and 2 differ in neuron density and UPR threshold: tissue 1 is a 3x3 neuron grid with a low UPR threshold ($\sigma_{1}=100$), and tissue 2 is a 5x5 neuron grid with a higher UPR threshold ($\sigma_{2}=300$).

**Figure 2:**
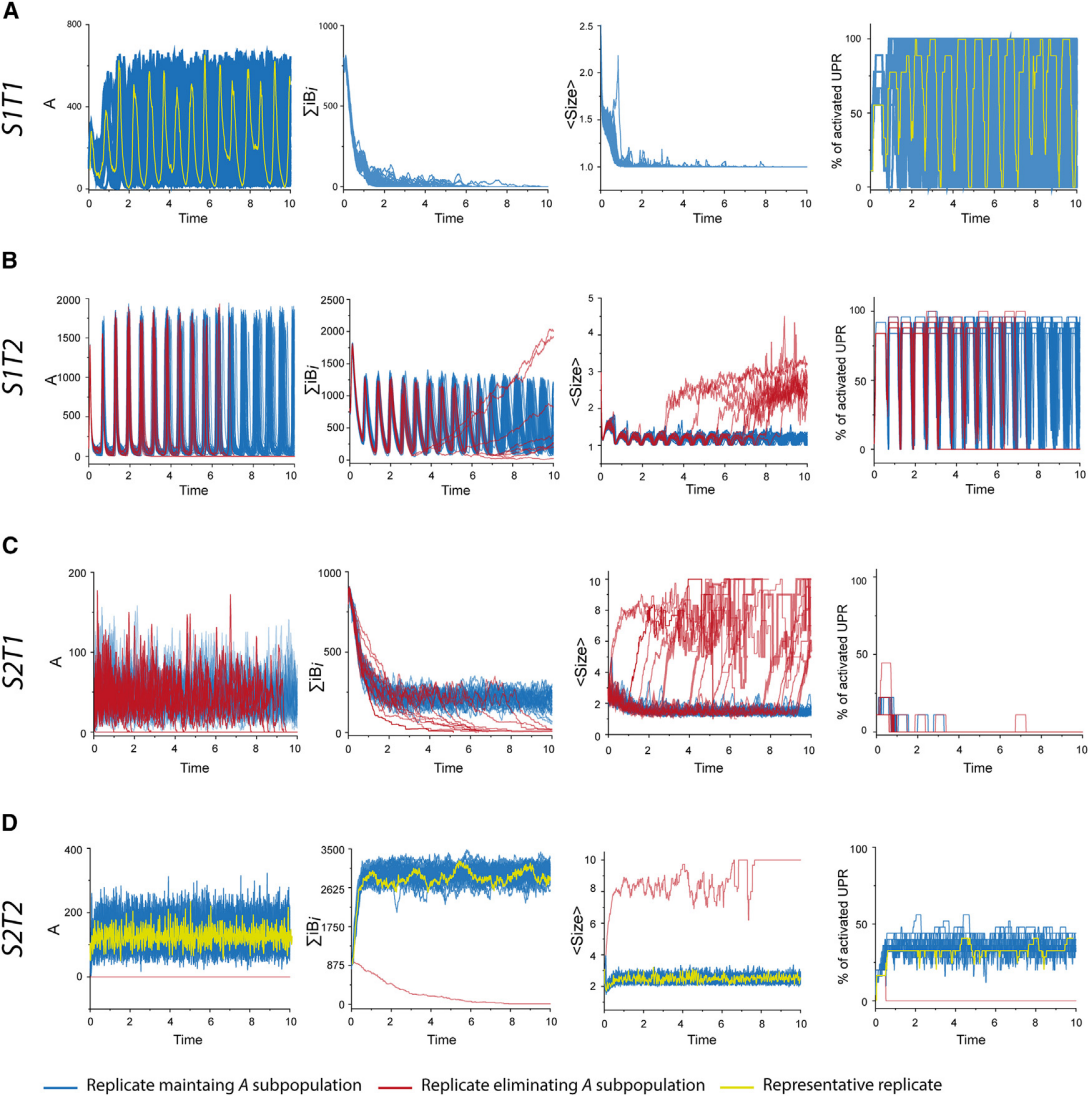
Evolution of strain 1 and strain 2 on two neurons-beds differing in neuron density and UPR activation threshold (see method details for more details) Evolution of different metrics characterizing the process of replication and its sustainability: *A*, $\sum iB_{i}$, average size of assemblies (*<Size>*) and percentage of neurons with activated UPR. In all panels, replicates where population *A* was maintained within the simulation time frame are represented in blue while red curves correspond to replicates where *A* was eliminated before the end of the simulation. In rows (A) and (D) where almost all replicates converge, a typical evolution is highlighted in yellow. (A) and (B) correspond to the evolution of strain 1 on tissue 1 (*S1T1*) and tissue 2 (*S1T2*) respectively, while (C) and (D) represent the evolution of strain 2 on tissue 1 (*S2T1*) and tissue 2 (*S2T2*) respectively. For each of the four strain/tissue combinations, 50 independent replicates were computed.

**Figure 3:**
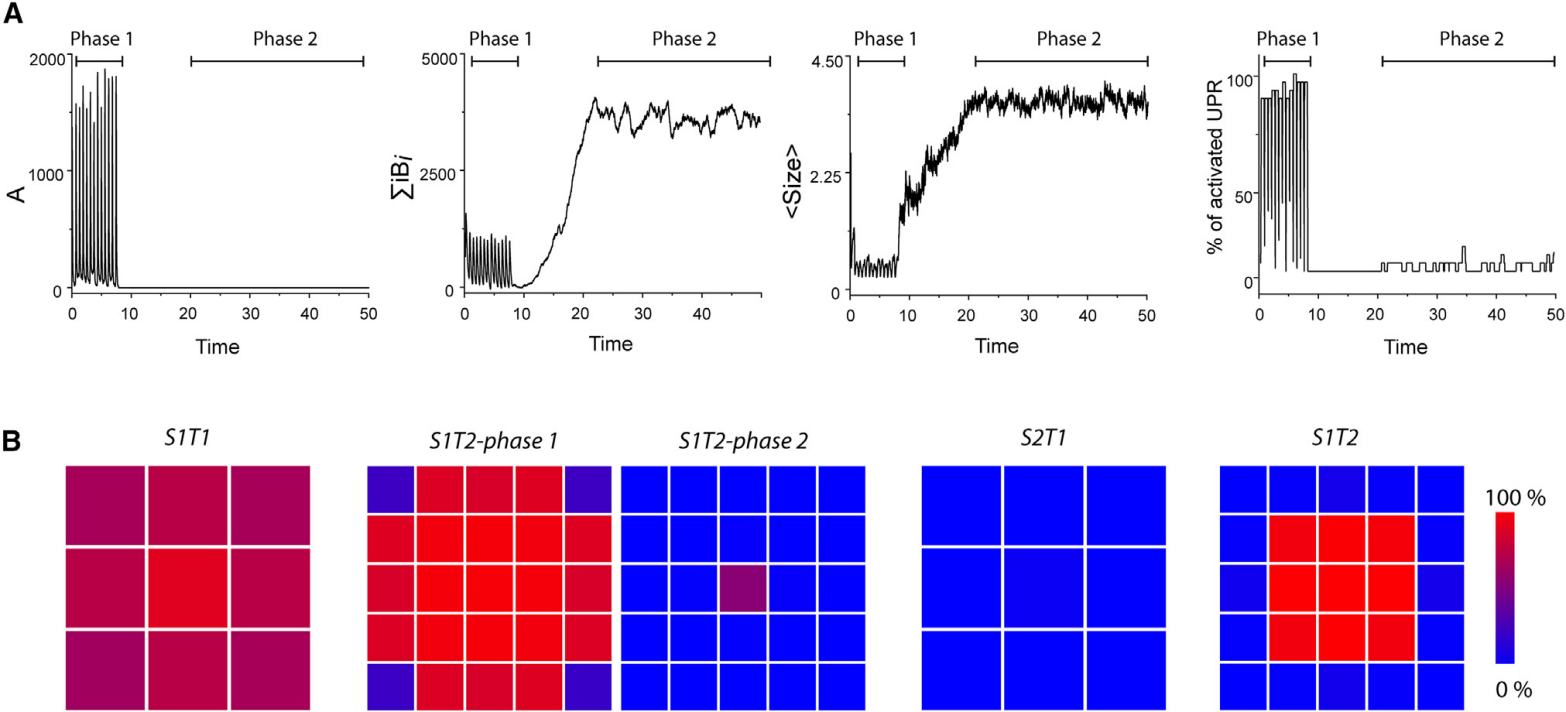
Extension of temporal replicates and spatial pattern evaluation (A) An extension of one replicate of *S1T2* (Figure 2B) with the same metrics highlighting the bifurcation between the two states depending on the elimination of *A*. The two phases are indicated above each panel. (B) Spatial pattern of the average percentage of simulation time spent in UPR activated state for each neuron over fifty independent replicates. (*S1T1*) and (*S2T1*) correspond, respectively, to the evolutions of strain 1 and strain 2 on tissue 1, which is a 3x3 neuron grid. (*S1T2-phase1*/*S1T2-phase2*) and (*S2T2*) correspond, respectively, to the evolutions of strain 1 and strain 2 on tissue 2, which is a 5x5 neuron grid. *S1T2* has two patterns corresponding to the two equilibrium phases previously described. The first one (*S1T2-phase1*) is averaged between the start of the simulation and the moment *A* is eliminated. The second pattern (*S1T2-phase2*) is obtained by averaging the signals from the first UPR activation after *A* has been eliminated to the end of the simulation.

Computational analysis of strain 1 on tissue 1 (*S1T1*) revealed the systematic elimination of the *B*_*i*_ subpopulation after a short transient phase, while the remaining *A* assemblies exhibited oscillatory behavior (Figure 2A and Video S1). The tissue response to the sustained evolution of strain 1 was characterized by periodic, synchronized UPR pulses across all neurons. However, neurons closer to the center of the grid spent more time in an activated state Figure 3B). The consistent reproducibility of these observations over fifty independent simulations suggests a convergence in the evolution of *S1T1*, leading to a stable equilibrium.

Video S1. Simulation video of strain propagation on a homogeneous neuron bed for S1T1, related to Figure 2 Three metrics (*A*, $\sum iB_{i}$, UPR) are graphed on top with animated vertical time-lines indicating at each instant the corresponding moment represented in the video under. Neurons are displayed as crosses, colored in white when their UPR is inactive and pink otherwise. The assemblies are represented as colored dots, *A* in green and *B*_*i*_ in a red to yellow gradient based on size (small in red and large in yellow).

In contrast, when strain 1 was applied to tissue 2 (*S1T2*), the results differed, highlighting the influence of tissue on prion assembly evolution. While *B*_*i*_ assemblies were rapidly eliminated in *S1T1*, *S1T2* displayed a transient equilibrium where both *A* and *B*_*i*_ subpopulations coexisted (Figure 2B and Video S2). This state appeared unstable, as the proportion of replicates eliminating their *A* subassembly increased with simulation time (Figure S1). Once the *A* population was eliminated, the *B*_*i*_ assemblies increased in both quantity and size, eventually reaching what appeared to be a new stable steady state (Figures 2B and 3A). During the transient state where *A* and *B*_*i*_ coexisted, an out-of-phase oscillatory behavior driven by replication and UPR activation was observed in both subpopulations. Unlike in *S1T1*, however, the UPR of the neurons in *S1T2* were not synchronized (Figures 2B and 3B). Notably, the average activation gradient in tissue 2 was much steeper, with central neurons occasionally failing to deactivate, while corner neurons were less frequently triggered (Figure 3B). Following the elimination of the *A* population, the UPR signal deteriorated, only reactivating after the new equilibrium was established, and confining to a few central neurons (Figure 3B).

Video S2. Simulation video of strain propagation on a homogeneous neuron bed for S1T2, related to Figures 2 and 3 Three metrics (*A*, $\sum iB_{i}$, UPR) are graphed on top with animated vertical time-lines indicating at each instant the corresponding moment represented in the video under. Neurons are displayed as crosses, colored in white when their UPR is inactive and pink otherwise. The assemblies are represented as colored dots, *A* in green and *B*_*i*_ in a red to yellow gradient based on size (small in red and large in yellow).

The evolution of strain 2 on tissue 1 (*S2T1*) exhibited more chaotic behavior compared to *S1T1* (Figure 2C and Video S3). The *A* and *B*_*i*_ subpopulations coexisted transiently in roughly constant quantities, similar to those in *S1T1*. However, this equilibrium appeared unstable and led to the elimination of species *A* (Figure S1). Following this event, the *B*_*i*_ subpopulation increased temporarily in size but was gradually eliminated, resulting in abortive replication within the simulation timescale. Interestingly, despite this transient replication, *S2T1* induced minimal UPR activation in the neurons (Figures 2C and 3B).

Video S3. Simulation video of strain propagation on a homogeneous neuron bed for S2T1. related to Figure 2 Three metrics (*A*, $\sum iB_{i}$, UPR) are graphed on top with animated vertical time-lines indicating at each instant the corresponding moment represented in the video under. Neurons are displayed as crosses, colored in white when their UPR is inactive and pink otherwise. The assemblies are represented as colored dots, *A* in green and *B*_*i*_ in a red to yellow gradient based on size (small in red and large in yellow).

In tissue 2, strain 2 (*S2T2*) displayed a similar pattern of accumulation compared to *S2T1*, with stable quantities of *A* and *B*_*i*_ assemblies, though the *B*_*i*_ subpopulation was favored (Figure 2C and Video S4). The average size of the assemblies was significantly larger. *S2T2* triggered UPR, but unlike *S1T2*, there were no oscillations. Notably, the UPR was almost continuously active in central neurons, while edge neurons showed minimal activity (Figure 3A). This behavior was consistently observed in nearly all replicates (49/50).

Video S4. Simulation video of strain propagation on a homogeneous neuron bed for S2T2. related to Figure 2 Three metrics (*A*, $\sum iB_{i}$, UPR) are graphed on top with animated vertical time-lines indicating at each instant the corresponding moment represented in the video under. Neurons are displayed as crosses, colored in white when their UPR is inactive and pink otherwise. The assemblies are represented as colored dots, *A* in green and *B*_*i*_ in a red to yellow gradient based on size (small in red and large in yellow).

**Figure 4:**
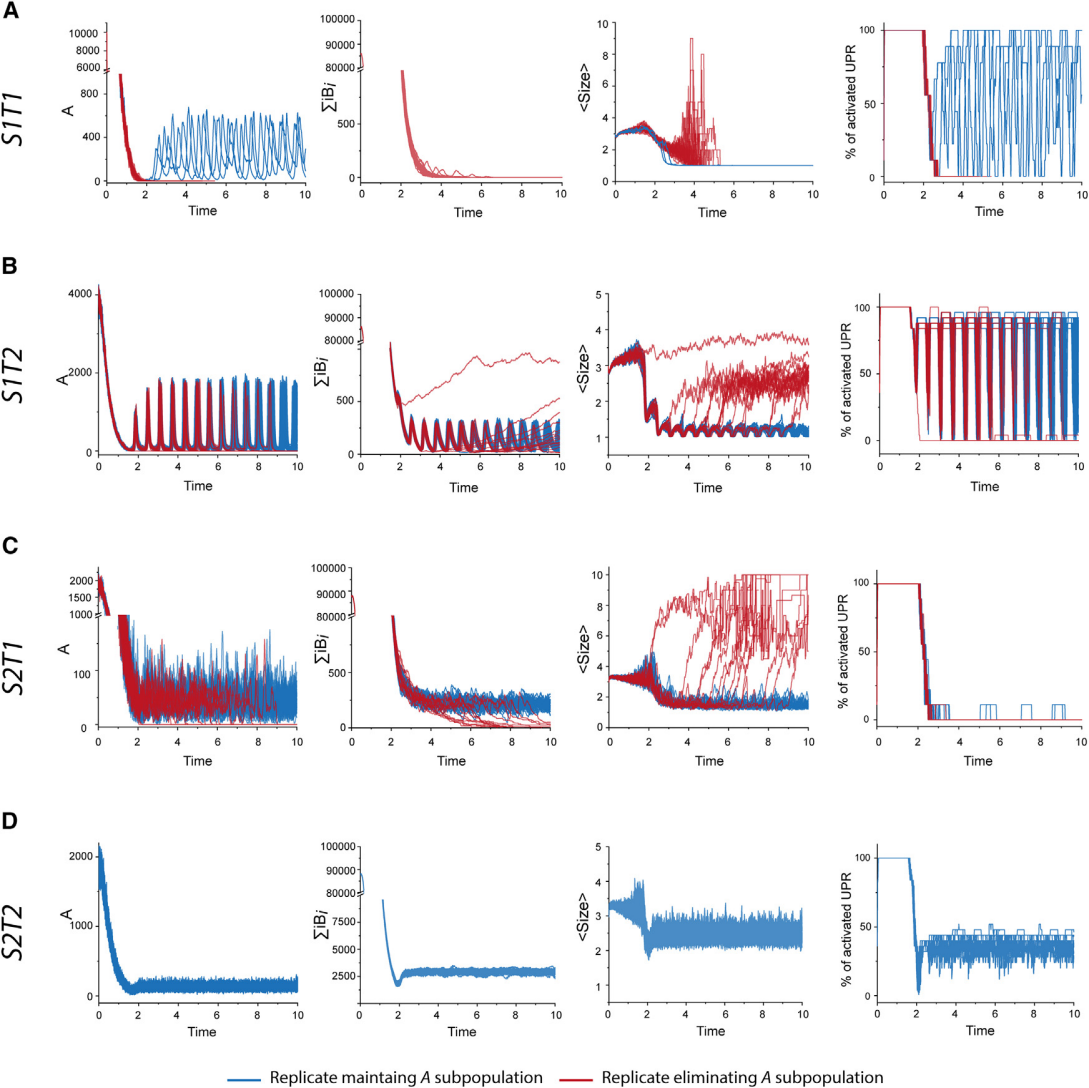
Effect of the initial seed amount on the evolution of strain 1 and strain 2 on tissue 1 and tissue 2 As in Figure 2, the evolutions of *A*, $\sum iB_{i}$, average size of assemblies (*<Size>*) and percentage of neurons with activated UPR are represented for 50 independent replicates. (A) and (B) correspond to the evolution of strain 1 on tissue 1 (*S1T2*) and tissue 2 (*S1T2*) respectively, while (C) and (D) represent the evolution of strain 2 on tissue 1 (*S2T1*) and tissue 2 (*S2T2*) respectively. Compared to the results on Figure 2, increasing the seed amount does not appear to impact the final equilibriums but, for *T1S1*, most replicates eliminate their *A* subassemblies and fail to reach the outcome previously observed. The comparison between initial seeding conditions can be further analyzed with the help of Figure S1 showing the percentage of replicates maintaining their *A* subassemblies as a function of simulation time.

We also investigated the impact of initial seeding concentration on system evolution by repeating the 50 replicates for each of the four strain-tissue combinations, increasing the initial assembly quantity 100-fold. Interestingly, only *S1T1* simulations were affected by this change. In the other three combinations (*S1T2*, *S2T1*, and *S2T2*), the system’s evolution remained consistent with that observed under nominal initial conditions (Figure 4). After brief transient phases where UPR remained activated, these simulations converged to their respective behaviors under standard conditions (Figure 2). Specifically, *S1T2* and *S2T1* maintained seemingly unstable states, leading to the selection of subpopulation *B*_*i*_ or the elimination of both subpopulations, respectively, while *S2T2* replicates reached the same equilibrium as under nominal conditions (see Figure S1 for comparisons between initial seeding conditions). In *S1T1*, the increased initial quantity caused most replicates (47/50) to eliminate their *A* subpopulation, followed shortly by the *B*_*i*_ assemblies. Interestingly, the three replicates that deviated from this pattern reached the same equilibrium as under nominal conditions.

Our results demonstrate that prion replication dynamics are significantly influenced by both strain-specific kinetics and local tissue properties. Variations in strain-tissue combinations led to diverse behaviors, including different patterns of UPR activation and equilibrium states. The initial quantity of assemblies impacted transient dynamics but did not alter the long-term equilibrium for most combinations. These findings elucidate the complex interplay between prion strains and tissue environments in shaping prion pathology.

### Co-propagation of two strains

The dynamic interplay between PrP^Sc^ assemblies and tissue response influences the sustainability of prion replication and the selection PrP^Sc^ subassemblies. This raises the question of how tissue response might affect the co-propagation of two strains with different templating activities, as seen in prion field isolates and affected mammalian species.^42^^,^^47^ To investigate this, we adapted the previous framework, which computed the evolution of prion assemblies on a homogeneous neuron bed, to include two strains. Although kinetically independent, both strains equally contribute to activating the UPR in neurons. The UPR is triggered when the combined assembly levels of the two strains exceed the threshold.

**Figure 5:**
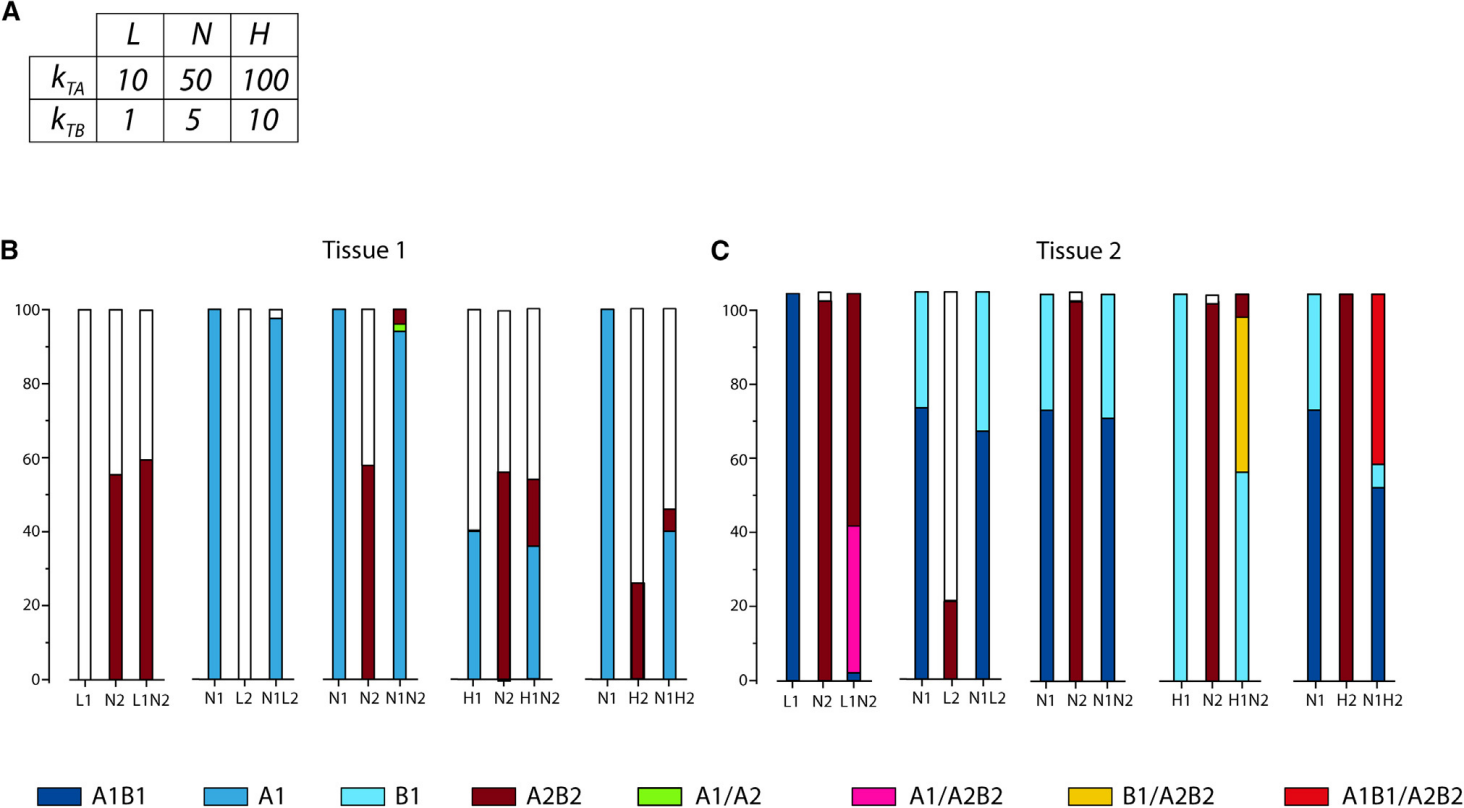
Effect of the replication rate on the evolution and co-propagation of strains on tissues 1 and 2 (A) By taking as a reference the templating parameters of strains 1 and 2 described in Figure 1B (named *N1* and *N2* respectively), we either doubled the replication rates for both *A* and *B*_*i*_ (*H1* and *H2* for strain 1 and strain 2 respectively) or divided them by 5 (*L1* and *L2* for strain 1 and strain 2 respectively) to get the high and low templating configurations for both strains. The evolutions of five different strain combinations were then computed and summarized on graph (B) for simulations on tissue 1 and graph (C) for tissue 2. The co-propagation of a strain combination is grouped with the two strains individually for ease of comparison. The bars represent the outcomes of 50 independent replicates, white means no assembly sustainably replicated within the simulation time frame.

Using the simulation parameters from the previous section for tissue 1 and tissue 2 (Figure 1F), we computed the co-propagation of strain 1 and strain 2 (Figure 1B), varying their templating rates. The original templating rates were labeled as nominal (*N*), with additional lower (*L*) and higher (*H*) rates introduced (Figure 5A). We compared the evolution of several combinations with the respective strains individually to study their interference under co-propagation.

As shown in the previous section, replication of strain 1 and strain 2 in tissue 1 with nominal templating rates (*N1* and *N2*) resulted in sustainable replication in 100% and 58% of the replicates, respectively. For strain 1, *B*_*i*_ assemblies were eliminated, whereas strain 2 maintained both subpopulations in the sustainable replicates (Figures 2A, 2C, and 5A). When the templating rate was lowered (*L1* for strain 1 and *L2* for strain 2), replication became unsustainable in 100% of the replicates for both strains (Figure 5B).

Under co-propagation conditions, where one strain had a nominal replication rate and the other a lower rate (*L1N2* and *N1L2*), the outcomes closely resembled those of the nominal strain alone. In the L*1N2* condition, 60% of the replicates showed the same results as 58% of the *N2*-alone replicates. In the *N1L2* condition, nearly all replicates (98%) resulted in the selection of subpopulation *A* from strain 1, similar to the results with *N1* alone. These L*1N2* and *N1L2* conditions illustrate a scenario where the tissue consistently favors the more effective replicator with minimal interference between the two strains.

With both templating rates set to nominal (*N1N2*) on tissue 1, strain 1’s *A* subpopulation was predominantly selected in 94% of the replicates, compared to 4% for strain 2. Interestingly, 2% of simulations resulted in sustained co-propagation of both strains. Despite sharing the same templating rates, the near-total selection of strain 1 in this scenario highlights the impact of strain-specific intrinsic dynamics of PrP^Sc^ assemblies during co-propagation.

Unexpectedly, increasing the templating rates (*H1* and *H2*) led to decreased sustainable replication for both strains on tissue 1 compared to their nominal rates, despite selecting the same subpopulations (40% for *H1* and 26% for *H2*; Figure 5B). In the co-propagating condition *H1N2*, strain 1 was predominantly selected in 36% of replicates, while strain 2 accounted for 18%. Although the proportion of replicates favoring strain 1 was similar to its evolution alone under *H1* conditions, the number of sustained strain 2 replicates decreased from 58% in *N2* to 18% in *H1N2*. This suggest that even though strain 1 had a higher templating rate (*H1*) but was less sustainable than strain 2 at nominal rates (*N2*), it was preferentially selected during co-propagation, at the expense of *N2*.

Moreover, while *N1* alone resulted in 100% sustained replication, combining it with *H2* (26% alone) led to less than half of the replicates showing sustained replication: the *N1H2* condition ended with 40% strain 1 selection and 6% strain 2 selection. This indicates that, despite lacking direct kinetic interaction, both strains exerted negative feedback on each other’s replication, resulting in fewer sustained evolutions when combined than when alone.

On tissue 2 (Figure 5C), strain 1 at nominal templating rate (*N1*) was maintained in 100% of the replicates, but with two distinct outcomes: in 74% or replicates, both *A* and *B*_*i*_ subassemblies persisted, while in the remaining 26%, only the *B*_*i*_ subpopulation remained. Replication of strain 2 at nominal rates (*N2*) on tissue 2 led to 98% of replicates maintaining both subassemblies. Similar to tissue 1, reducing strain 2’s templating rate (*L2*) significantly decreased the number of sustainable replicates (20%) compared to the *N2* condition. In the low-rate configuration, strain 1 (*L1*) was still maintained in 100% of simulations, but unlike in *N1*, both *A* and *B*_*i*_ subpopulations persisted in every case.

Co-propagation of strain 1 and strain 2 with low and nominal templating rates, respectively (*L1N2*), on tissue 2 yielded different outcomes compared to tissue 1. Notably, strain 1 was eliminated in 60% of the replicates, yet sustained co-propagation of both strains occurred in 38% of replicates, despite their different templating activities. Additionally, in replicates that maintained both strains, strain 1’s equilibrium shifted, maintaining only its *A* subassembly in the presence of both *A* and *B*_*i*_ subpopulations from strain 2. This illustrates that strains can influence each other during co-propagation, even without kinetic linkage, altering the balance of subpopulations.

The *N1L2* configuration on tissue 2 behaved similarly to tissue 1, with strain 1 dominating and minimal interference from strain 2, replicating the outcomes observed in the *N1* condition. This pattern held true even in the *N1N2* configuration, despite strain 2 sustaining evolution in 98% of replicates alone. When strain 2’s templating rate was increased (*H2*), it showed little deviation from its nominal behavior (*N2*) on tissue 2, as evidenced by the retention of both *A* and *B*_*i*_ subassemblies in all replicates (Figure 5C). While strain 1 was sustained in 100% of simulations at a high templating rate (*H1*) on tissue 2, only its *B*_*i*_ subassemblies replicated sustainably, unlike in the *N1* and *L1* configurations.

In co-propagating conditions involving highly replicative strains (*H1N2* and *N1H2*), sustainable co-propagation of both strains occurred in roughly equal proportions. Under the *H1N2* condition, 54% of replicates involved strain 1’s *B*_*i*_ subassemblies, 6% maintained both subpopulations of strain 2, and 40% exhibited sustained co-propagation of strain 1’s *B*_*i*_ subassemblies alongside both subpopulations from strain 2. In contrast, in the *N1H2* configuration, despite strain 2’s higher templating rate, it was not selected over strain 1 in any replicate. Interestingly, although strain 1 showed two possible outcomes when alone (both subassemblies persisting or *A* being eliminated in *N1*), co-propagation resulted in 48% of replicates maintaining both subassemblies of strain 1 consistently.

Throughout these simulations, we observed that the tissue plays a critical role in mediating interactions between strains. Even in the absence of direct kinetic interactions, the strains influenced each other through their equal contribution to the UPR, sometimes resulting in dominance-negative interference (e.g., N1H2 on tissue 1) or sustained co-propagation with specific subassembly selection. The differences in tissue parameters also impacted co-propagation outcomes, with tissue 2 supporting the coexistence of both strains, unlike tissue 1, which even failed to maintain highly replicative strains. Additionally, because of strain dynamics, we observed instances where the less effective replicator was selected, or where strains with different templating activities co-propagated sustainably.

### Contribution of the connectome to spreading

Our initial focus was on understanding how a modeled brain region contributes to the replication and selection of PrP^Sc^ assemblies. We then extended our study to explore the influence of the axonal neural network on the dissemination of these assemblies across three distinct strains (Figure 1B). Although these strains share the same templating activities, their intrinsic dynamics favor the *B*_*i*_ subpopulation from strain 1 to strain 3.

To simulate this, we modeled two neuron clusters positioned at opposite corners of a square-shaped domain, connected unilaterally by axons (Figure 1D). The number and size of voxels in the domain were increased to represent longer distances. Simulations were initiated near either the emitting or receiving neurons to induce anterograde or retrograde spreading, respectively. We varied the number of axons from 1 to 20 under both anterograde and retrograde conditions and compared the results to dissemination in the absence of axons (Figure 1G). Prion replication around neurons (somas and axons) is regulated by the UPR, which is triggered when PrP^Sc^ concentration reaches a threshold near the soma (see method details and Figure 1E for details).

**Figure 6:**
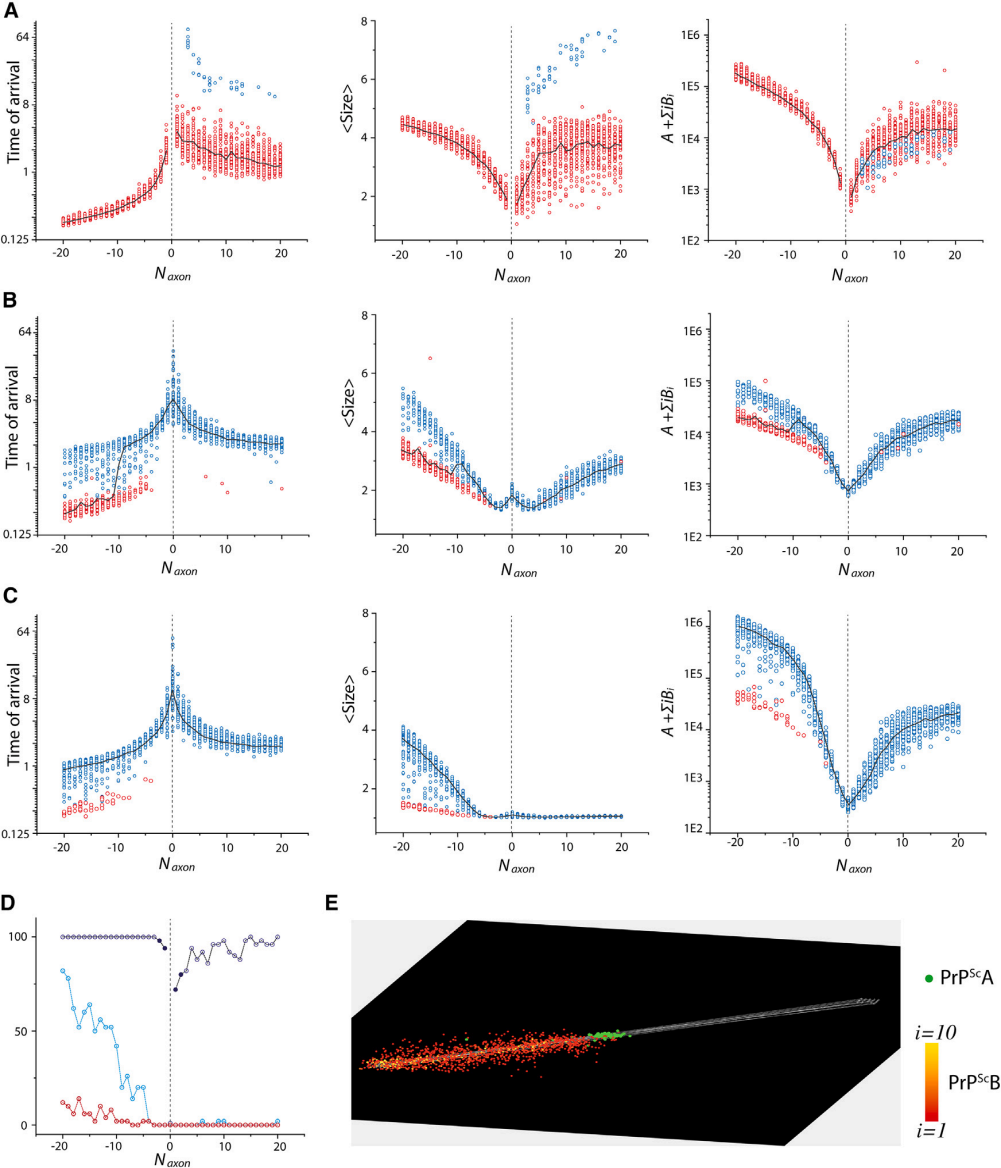
Influence of the number and orientation of axonal connections on the spreading process between two unilaterally linked neuron clusters for three distinct prion strains For all graphs, the x axis accounts for the number of axons (*N*_*axon*_) linking the clusters, negative values correspond to retrograde propagation (seeding at the receiving neurons) and positive ones to anterograde (seeding at the emitting neurons). For each connectivity value and each strain, fifty replicates were computed. Their output is represented by dots in the scatterplots of (A) for strain 1, (B) for strain 2 and (C) for strain 3. We studied the influence of connectivity on three different metrics: the time of arrival to the second cluster as well as the average size (<Size>) and quantity of assemblies ($A+\sum iB_{i}$) at the time of arrival. Blue dots correspond to simulations which eliminated their *A* subassemblies before reaching the second neuron group, while red ones maintained them. The black line is the median value of the replicates for each metric. (D) The proportion of replicates maintaining their *A* subassemblies during the simulation timescale as a function of connectivity (*N*_*axon*_) for all three strains highlights the impact of axons and UPR response on the sustainable replication of subpopulations. (E) Typical frame taken from a video of the retrograde dissemination process (see Video S5 for full video). Seeding was done at the receiving neurons on the left, *A* assemblies are represented in green while *B*_*i*_ assemblies are colored from red to yellow depending on size. This shows that the dissemination was guided by axons and facilitated by the *A* subpopulation being more replicative and diffusive. In this configuration, the system self-organized with a front of *A* followed by the *B*_*i*_ subpopulation, with larger assemblies located closer to the place of inoculation.

We monitored the dissemination of the three strains from one cluster to the other using three metrics: time of arrival at the opposing cluster (*t*_*arriv*_), average assembly size (*<Size>*), and quantity of assemblies ($A+\sum iB_{i}$) at *t*_*arriv*_ (Figure 6). Overall, the presence of axons reduced the time of arrival for both anterograde and retrograde pathways. Specifically, compared to dissemination without axons, both anterograde and retrograde connections significantly decreased *t*_*arriv*_ for all three strains (Figures 6A–6C). This effect was most pronounced for strain 1, which failed to reach the opposing cluster without axons (Figure 6A), suggesting that the connectome facilitates propagation by supporting replication along the axon’s path^48^ in both directions. Notably, retrograde spreading was faster on average than anterograde spreading for all three strains, likely because the UPR was not triggered until the second cluster was reached during retrograde dissemination, allowing unregulated replication along the axons.

Video S5. Simulation video of retrograde dissemination process between two neuron clusters. related to Figure 6 The two neuron clusters were located on opposite corners of the domain and unilaterally linked by axons (see Method details for more details). Seeding of the assemblies was done at the receiving neuron cluster. Neurons are represented as white crosses, axons as white lines. *A* assemblies are represented as green dots while *B*_*i*_ are colored using a red-yellow gradient to account for size, red corresponds to small *B*_*i*_ assemblies and yellow big ones. This video highlights the impact of replication on the dissemination process as templating of the assemblies occurred only in the vicinity of the axons. The role of both subassemblies was also brought out as the system spatially self-organized with a front of *A* assemblies, more replicative and diffusive, followed by a gradient of *B*_*i*_ assemblies, with larger objects located closer to the inoculation site.

During anterograde dissemination, strain 1 showed variability in maintaining its *A* subpopulation. Some replicates lost the *A* subpopulation by the time they reached the second neuron cluster, resulting in significantly higher *t*_*arriv*_ and larger assembly sizes compared to those that maintained *A*, though the quantity of assemblies remained similar (Figure 6A). As anterograde connectivity increased, more strain 1 replicates retained their *A* subpopulation during dissemination (Figure 6D). In contrast, strain 2 only occasionally maintained *A* during anterograde spreading, with no clear increase with connectivity (Figure 6D). These replicates showed lower *t*_*arriv*_ than those that eliminated *A*, but unlike Strain 1, this did not correspond to smaller or fewer assemblies (Figure 6B). Strain 3 consistently eliminated the *A* subpopulation before reaching the second cluster during anterograde spreading (Figure 6D). Although the *t*_*arriv*_ and quantity of assemblies evolved similarly to the other strains, strain 3 consistently favored small *B*_*i*_ assemblies during anterograde spreading, regardless of the number of axons (Figure 6C).

In retrograde dissemination, strain 1 uniformly maintained its *A* subpopulation (Figure 6D). Retrograde spreading was faster and resulted in more assemblies compared to anterograde, with a similar size distribution (Figure 6A). Strains 2 and 3 displayed dual behaviors during retrograde spreading, depending on whether the *A* subpopulation was maintained (Figures 6B and 6C). With low retrograde connectivity, all replicates of strains 2 and 3 eliminated *A* during dissemination, but the proportion retaining *A* increased with the number of axons, particularly for Strain 2 (Figure 6D). Maintaining *A* during retrograde spreading notably increased dissemination speed, reaching that of strain 1, though the average size and quantity of assemblies were lower (Figures 6B and 6C). Strain 3 showed the most significant differences based on spreading direction, accumulating larger assemblies more abundantly in the retrograde direction. These distinct dissemination patterns raise questions about the spatial distribution and evolution of *A* and *B*_*i*_ subpopulations when *A* is retained. As observed in the time-lapse spatiotemporal retrograde spreading of strain 1, a front of *A* assemblies preceded *B*_*i*_ subassemblies (Figure 6E; full movie in Video S5). This faster spreading of population *A* appeared to facilitate subsequent *B*_*i*_ dissemination, leaving a trail of larger and more numerous assemblies near the starting point.

In summary, these findings indicate that connectome density and the direction of spreading (anterograde or retrograde) interact with strain properties to determine the speed, size, and number of prion assemblies during dissemination. The retention of *A* subassemblies, influenced by connectivity and strain kinetics, plays a critical role in this process.

## Discussion

Understanding the link between the replication and dissemination of prion assemblies through brain tissue is crucial for elucidating prion pathologies. However, this relationship remains poorly understood, as PrP^Sc^ deposition patterns, neuronal loss, and gliosis in prion diseases substantially differ from those observed in other proteinopathies. While the brain’s connectome plays a central role in the pathogenesis of Alzheimer’s and Parkinson’s diseases,^17^^,^^18^ its contribution to PrP^Sc^ deposition patterns is still unclear.^19^ The progression of prion diseases and the dissemination of prion assemblies appear to be more complex and strain-dependent compared to other related pathologies.

Extensive modeling approaches have been developed to link the prion-like aggregation mechanisms of amyloid beta, tau, and alpha-synuclein with their spread through the brain’s connectome.^47^^,^^48^^,^^49^^,^^50^^,^^51^ These models typically share common features: linear replication via end-elongation, fragmentation to increase the number of templating interfaces,^51^ and directed spreading pathway through the structural connectome, in line with Braak’s directional hypothesis.^52^ However, most of these models fail to account for the coexistence of multiple subpopulations, such as the small oligomeric Aβ assemblies and the various types of Aβ fibrils observed in Alzheimer’s disease. In the context of mammalian prion diseases, similar modeling approaches are almost nonexistent, especially those that describe the spreading process, strain-specific patterns, strain coevolution, or strain-tissue tropisms.^19^ When such models do exist, they often rely on the same features that may not be fully applicable to prion pathologies, such as intra-axonal transport, despite PrP^Sc^ assemblies being extracellular.

**Figure 7:**
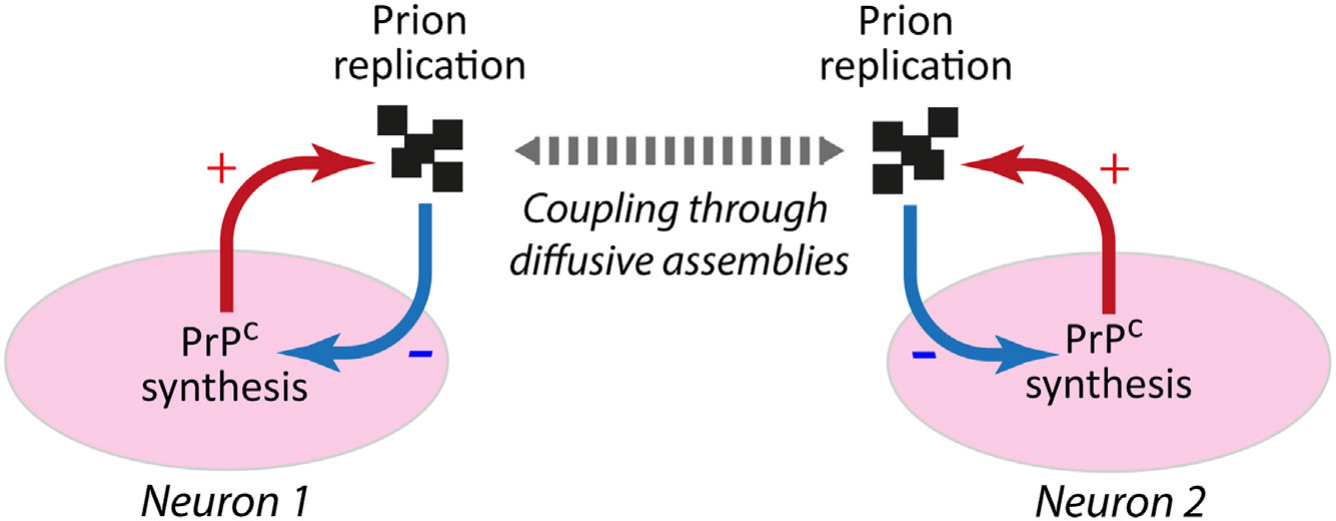
The interplay between prion replication and tissue response is a complex feedback loop Misfolded PrP assemblies accumulate near neurons due to the replication process, leading to downregulation of PrP^C^ via UPR activation. This mechanism, combined with the extracellular diffusion of prion assemblies, whether via Brownian motion or guided diffusion along axons, drives prion dissemination. It may also contribute to the coupling of cell responses within a brain region, influencing the selection of specific types of PrP^Sc^ subassemblies and the emergence of spatial PrP^Sc^ deposition patterns.

The model developed in the present study builds on recent advancements in understanding prion replication mechanisms. It incorporates multiple subpopulations engaging in autocatalytic processes and includes a simplified tissue response to prion propagation. This response is modeled as a negative feedback mechanism that impacts PrP^C^ production via the unfolded protein response (UPR). By biochemically formalizing prion strains as a convolution of the intrinsic dynamics of PrP^Sc^ assemblies and their replicative properties, we demonstrated that PrP^Sc^ replication, accumulation, and neuronal response within a brain area depend non-linearly on both strain and tissue properties. This interplay between the dynamic nature of PrP^Sc^ assemblies and the tissue response influences the sustainability of the replication process and the co-propagation of different PrP^Sc^ subpopulations or distinct strains with varying templating activities in coinfection conditions. In this work, we also propose a valid alternative to step-by-step spreading through the structural connectome: extracellular diffusion coupled with replication in the vicinity of neurons (Figure 7).

The tissue response is an integral part of the prion replication process. The modification of the structural and functional neural networks, neuronal death, astrocytosis, UPR activation, and metabolic response are all part of the complex tissue response to prion replication.^53^ Despite this complexity, we chose to focus solely on the UPR in this work to simplify the model, as it is one of the earliest responses to prion propagation and its mechanisms have been extensively studied.^35^^,^^36^^,^^37^^,^^38^^,^^39^ It has been reported that the accumulation of PrP^Sc^ locally triggers the UPR in neurons, resulting in the downregulation of proteins transiting through the endoplasmic reticulum, including PrP^C^^39^^,^^54^. This negative feedback of PrP^Sc^ on PrP^C^ production can interfere with prion propagation by introducing non-linearity into the replication process.^54^ Considering other types of tissue responses or a combination of them could increase the non-linearity of the response to prion replication, thereby adding further complexity to the system and allowing for the emergence of new behaviors.

The tissue response to PrP^Sc^ accumulation is known to be cell-specific. Different types of neurons may exhibit varying thresholds of UPR activation, with downstream effects depending on the cellular context.^55^^,^^56^ This variability makes the UPR a suitable candidate for a local tissue parameter. In our model, the interruption of templating due to the accumulation of assemblies can also be interpreted as PrP^C^ becoming the limiting factor in the replication process once PrP^Sc^ concentration reaches a certain threshold. This would also qualify as a local tissue parameter, as the latency in PrP^C^ production could fluctuate between cell types, causing similar non-linearities.

As typically shown in the evolutions of *S1T1* and *S1T2*, the oscillatory behavior of replication is a direct consequence of the negative feedback of UPR on PrP^C^ production. However, the absence of periodic patterns in the accumulations of *S2T1* and *S2T2* demonstrates that UPR is not the sole factor involved. In our simulations, oscillations in PrP^Sc^ quantity systematically occur under conditions where the proportion of *A* subassemblies is significant, as observed in *S1T1* and *S1T2*. In these situations, the tissue’s response to accumulation involves synchronized UPR firings of the neurons. Conversely, simulations with higher proportions of *B*_*i*_ subassemblies do not exhibit oscillatory behaviors in PrP^Sc^ quantity or UPR signals. Notably, in the case of *S2T1*, there is even a temporary state where replication does not elicit any tissue response.

While the high replicability and diffusivity of *A* induce synchronization among all neurons, the inertia caused by the lower diffusivity and condensation of *B*_*i*_ subassemblies decreases the coupling distance. This results in continuous stimulation of neurons in the middle of the grid, thereby causing the emergence of spatial patterns. As the balance between *A* and *B*_*i*_ subpopulations partially depends on the strain, this indicates that PrP^Sc^ accumulation results from an interplay between the tissue response and the intrinsic dynamics of strain replication.

As demonstrated by propagation simulations *S2T1* or *S1T2*, we found instances of stable or transient unstable replication during which the UPR is barely triggered, if at all. Although the tissue response in this model is highly simplified and restricted to the UPR, this behavior suggests that silent replication, which escapes the tissue response, could occur with certain combinations of tissue parameters and strain dynamics.

Interplay between PrP^Sc^ dynamics and tissue response governs tissue tropisms, selection of subassemblies, and co-propagation. It is commonly believed that the initiation and sustainability of prion replication in a given tissue are governed by the compatibility between PrP^Sc^ assemblies and the local conformome of PrP^C^ or local cofactors that thermodynamically influence the homotypic replication process. Our simulations revealed that introducing non-linearity in the form of a tissue response to prion replication, which modulates PrP^C^ expression, could impact both the sustainability of the replication process and the selection of PrP^Sc^ subassemblies. As shown in Figures 2 and 3, with limited strain-tissue parameter combinations, we observed a wide variety of outcomes, such as the selection of a specific PrP^Sc^ subpopulation depending on the tissue (*S1T1* vs. *S1T2*) or transient unstable replication (*S2T1*). We also demonstrated that for some strain-tissue combinations, increasing the replication rate (Figure 4) or the initial seed concentration can negatively impact the sustainability of the replication process, as highlighted by the behavior of *S1T1* (Figures 2 and 3). These observations suggest that the resonance between a non-linear tissue response and the inherent dynamic nature of prion assemblies could explain strain-specific tissue tropisms, providing a viable alternative to the local conformome hypothesis.

Our simulations revealed the existence of transient replication regimes. The evolutions of *S1T2* and *S2T1* show initial states where both subpopulations are transiently maintained. However, both these equilibria appear unstable, eventually leading to the elimination of *A* subassemblies as simulation time increases (Figure S1). Due to the highly dynamic nature of the kinetic scheme, the elimination of one subpopulation results in a drastic change in behavior. For *S2T1*, the loss of subpopulation *A* is shortly followed by that of *B*_*i*_, resulting in abortive replication, while *S1T2* reaches a new equilibrium composed solely of *B*_*i*_ subassemblies. Despite similarities, the two transient replication regimes elicit radically different responses from neurons: *S1T2* presents periodic firing of the UPR in most neurons, while *S2T1* provokes no response. This shows that transient replication relies on both strain dynamics and tissue-modulated replication, highlighting this interplay as a key factor in prion propagation. These transient replication regimes could explain experimentally observed non-adaptive prion amplification.^57^

The non-linearity introduced in our model by the tissue response plays a key role in the co-propagation of two prion strains within the same brain area. We showed that the co-propagation of two strains can alter their respective evolution or sustainable replication, giving rise to negative dominance interferences.^58^^,^^59^ Because the strains are kinetically independent in our model, this interference is a direct result of the competition for the same substrate and equal contribution to the UPR of the neurons, highlighting the influence of the tissue response in the strain selection process.

The tissue also appears to play a crucial role in the coexistence of two strains. As shown in Figures 5B and 5C, tissue 1 does not allow the sustained co-propagation of any combination of our two modeled strains, while tissue 2 does. This type of behavior is observed experimentally in prion field isolates, where two strains can coexist within the brain, while other organs, such as the spleen, only maintain one of them.^60^

Combined with the non-linear tissue response, the intrinsic dynamic nature of prion assemblies, reflected in the kinetic scheme comprising several autocatalytic reactions between different subpopulations, allows for the possible elimination of the best replicator or the sustained co-propagation of strains with different templating activities. This provides answers to strain selection, co-propagation, and negative dominance effects that are not based on hypothetical cofactors or structural compatibility between PrP^Sc^ and tissue-specific PrP^C^ conformers, which are thermodynamic considerations, but on kinetic considerations and complex system responses.^61^

The connectome contributes to the spreading process by increasing the dissemination speed and exerting selection pressure on assemblies. One of the main questions in the field of prions is how the connectome contributes to the spreading and neuro-invasion processes. Experimental observations have clearly highlighted the impact of both peripheral anterograde and retrograde connections^62^^,^^63^ while excluding axonal cytoskeleton transport.^63^ In our simplified configuration, featuring two groups of neurons linked unilaterally, we demonstrated that axons facilitate prion dissemination and promote the replication of assemblies, increasing both their quantity and average size.

Unlike previous works that assumed axonal transport, our model presents an alternative, where the assemblies are simply guided by the connectome. They disseminate according to Brownian motion and can replicate in the vicinity of axons. In this context, despite different tissue configurations and kinetic parameters, the sustainability of *A* subassemblies once again emerged as a key factor in the spreading process. It acts as a switch between two distinct regimes, notably differing in propagation speed. If subpopulation *A* is maintained during propagation, for a given strain and connectivity, the dissemination speed increases significantly, while the quantity and size of assemblies at the time of arrival are reduced.

This facilitating role is due to subpopulation *A*’s high diffusivity and replicability, combined with its crucial role in the highly catalytic kinetic scheme. This is reflected in the spatial organization of assemblies during the dissemination process: a front of *A* followed by a gradient of *B*_*i*_ subassemblies, with smaller *B*_*i*_ objects trailing due to *B*_*1*_ being a predator of *A* (Video S5). Consequently, the retention of *A* by the time the second neuron group is reached depends on the intrinsic dynamics of the strains as well as the connectivity, with axons contributing to the sustainability of *A*, especially in retrograde spreading.

Due to the progressive shift in behaviors occurring as retrograde connectivity increases, we once again highlighted a resonance between tissue and strain parameters, resulting in non-linearities in propagation speed and accumulation. Simulations also revealed a potential role of the connectome as a filter in the spreading process, selectively determining which types of assemblies propagate. This selection process could determine which assemblies initiate replication in the next region.

Additionally, one of the main conclusions emerging from this study is the difference between retrograde and anterograde dissemination. In all our simulations, strains propagate faster in the retrograde direction, a phenomenon exacerbated at high connectivity due to the previously highlighted shift in regimes primarily occurring in the retrograde direction. Strains also appear to react differently to the direction; for instance, the quantity of assemblies accumulated for strain 2 seems relatively independent of direction, while strain 3 accumulates significantly more. The difference in propagation speed depending on direction is not experimentally documented and could be a direct consequence of our modeling of the UPR. In our retrograde propagation model, the UPR associated with the axons is not triggered until the second group of neurons is reached (see method details), resulting in unregulated replication that greatly accelerates the dissemination process. Conversely, in the anterograde direction, the presence of aggregates around the somas of the first group of neurons immediately prevents replication along the axons. While it is possible that the assemblies moving up the axons would not elicit any tissue response, the replication process would be expected to decline due to the high number of assemblies causing the production of PrP^C^ to become the limiting factor. This would not impact the dissemination front, so we can expect the speed to be affected less than the quantity or size of assemblies.

In this study, we demonstrated that the interplay between tissue response non-linearities and highly dynamic prion kinetics renders prion propagation in brain tissue a complex system. This complexity arises from the interaction between negative feedback on PrP^C^ expression and multiple catalytic processes among different PrP^Sc^ subpopulations, leading to unpredictable behaviors such as specific subpopulation selection, interference between strains with negative dominance, and abortive, transient, or silent replication. Our findings offer an alternative explanation to the hypothetical variable compatibility between strains and local conformers of PrP^C^ for strain-specific phenotypes and tissue tropisms, which canonical linear models of prion replication (end-elongation or nucleation-elongation processes) fail to resolve.

Finally, this work sheds new light on the role of the connectome. While active transport significantly influences the dissemination of aggregation seeds in prion-like pathologies such as tauopathies and synucleinopathies,^18^^,^^52^ it does not substantially contribute to prion propagation and dissemination within tissue.^63^ Our simulations clarify the connectome’s role in dissemination, suggesting it could operate through extracellular guided diffusion and replication at the axons’ vicinity. This interaction with highly dynamic prion kinetics results in non-linear behaviors in dissemination speed, accumulation, and spatial organization of the assemblies.

### Limitations of the study

This work is based on a unique kinetic scheme that features experimentally observed structural diversification during prion replication and intrinsic dynamics of PrP^Sc^ assemblies, but the tissue response has been simplified to an all-or-nothing replication with the UPR, omitting crucial cell diversity. Consequently, our model does not account for gliosis or neuronal death, which exert positive and negative feedbacks on PrP^C^ levels, respectively, and may significantly impact the sustainable replication of specific prion strains and the selection of particular PrP^Sc^ subassemblies. This work highlights the local variation of PrP^C^ expression during pathogenesis as a critical parameter influencing infection sustainability. Although a global decrease in PrP^C^ expression has been reported during prion infection,^64^ the biochemical origin of this decrease remains unknown. Approaches that elucidate the spatiotemporal variation of PrP^C^ expression during tissue invasion will enable the parameterization of the tissue response.

Our model can approximate a local brain area with varying densities of a specific cell type or a primary neuron culture replicating prion,^65^ but it does not account for local physical factors like tissue rheology and tortuosity that could influence the diffusivity of assemblies and select specific PrP^Sc^ subpopulations. Moreover, the intricate kinetics between different populations challenges the experimental assessment of reaction constants, necessitating a trial-and-error approach to identify interesting regimes, thus limiting the biochemical relevance. Despite these limitations, this work highlighted the role of PrP^Sc^A in the sustainability of the replication process and in the spreading regime. Quantifying PrP^Sc^A levels^33^ during *ex vivo* infection assays, such as in primary neuron cultures or organotypic brain slices, and assessing dissemination speeds on a retrograde or anterograde axonal network using microfluidic systems^66^ could validate the model through a reverse engineering approach.

## Resource availability

### Lead contact

Requests for further information and resources should be directed to and will be fulfilled by the lead contact, Human Rezaei (human.rezaei@inrae.fr).

### Materials availability

This study did not generate new unique reagents.

### Data and code availability

All original code has been deposited at Zenodo at [https://doi.org/10.5281/zenodo.13897049] as of the date of publication.
All data used in this study can be regenerated using the code referenced above and the parameters specified in the article.
Any additional information required to reanalyze the data reported in this paper is available from the lead contact upon request.

## Acknowledgments

This work was supported by grants from 10.13039/501100001665ANR (PrionDif, ANR-21-CE15-0011-01), the 10.13039/501100000781European Research Council (ERC Starting Grant SKIPPERAD, number 306321), the Ile-de-France region (DIM MALINF) and Metaprogram DigitBio (PrionDiff).

## Author contributions

Conceptualization, H.R., B.F., and L.P.M.; Methodology, H.R., B.F., A.I., V.B., D.M., and P.S; Software, B.F.; Formal Analysis, B.F.; Data Curation, B.F.; Writing – Original Draft, H.R. and B.F.; Writing – Review and Editing, H.R., B.F., L.P.M., A.I., V.B., D.M., and P.S.; Visualization, H.R. and B.F.; Supervision, H.R. and L.P.M.

## Declaration of interests

The authors declare that they have no known competing financial interests or personal relationships that could have appeared to influence the work reported in this paper.

## STAR★Methods

### Key resources table

**Table:** 

REAGENT or RESOURCE	SOURCE	IDENTIFIER
**Deposited data**		

Code used for simulations in this work	This paper	

**Software and algorithms**		

MATLAB_R2021a	MathWorks®	https://www.mathworks.com/products/matlab.html

### Method details

#### Dynamics of the coexistence of different PrP^Sc^ subpopulation and kinetic scheme of their replication

The kinetic model of prion assemblies used in this work is based on the recent evidence of structural diversification occurring during replication^33^ and the dynamic nature of different PrP^Sc^ subpopulations.^16^^,^^34^^,^^67^ Regardless of the strain, prion replication generates two structurally distinct sets of assemblies called PrP^Sc^A and PrP^Sc^B_i_ (from now on called respectively *A* and *B*_*i*_). The *A* and *B*_*1*_ species are both dimers of PrP differing in structure and *B*_*i*_ is a condensate of *B*_*1*_ where i represents the number of *B*_*1*_ elementary units it comprises i.e., its size.^33^ Both subassemblies have the ability to spread the strain information from which they are issued. While *B*_*i*_ grows by one unit during replication, *A* replicates by creating a copy of itself, its size remaining constant.^33^ As we consider that *B*_*i*_ assemblies replicate at their extremities, the number of templating interfaces remains the same regardless of their size, therefore the templating of *B*_*i*_ was chosen to be independent of size. Additionally, bioassay experiments proved that the specific infectivity (i.e., replication rate) of subpopulation *A* is substantially higher than that of *B*_*i*_.^33^

Biochemical characterization of different prion strains established the existence of a constitutional equilibrium between *B*_*i ≥ 2*_ and *B*_*1*_ assemblies.^16^^,^^67^ Based on oscillatory behavior observed during relaxation kinetics experiments, Mezache and colleagues completed this model with autocatalytic processes between *A* and *B*_*i*_ subpopulations.^34^ The complete kinetic model is reported in Figure 1A.

#### Templating field and tissue response to prion replication and accumulation

While PrP^Sc^ assemblies are extracellular, PrP^C^ is mostly located on the surface of cells attached by a glycosylphosphatidylinositol (GPI) anchor,^68^ templating reactions therefore predominantly occur in the vicinity of cells. In our model, replication of both *A* and *B*_*i*_ subassemblies can only happen inside a zone around neurons we call templating field (Figures 1C and 1D), inside which we consider PrP^C^ to be in excess and evenly distributed. On the surface of neurons, PrP^C^ is more abundant around the somas than along the axons.^69^ In our model, this difference translated into the templating field being wider around the somas than the axons.

As reported in the literature, prion accumulation in the vicinity of a cell induces the unfolded protein response (UPR), through either PrP^Sc^ endocytosis pathways^70^ or extracellular sensors,^35^ which leads to the down regulation of PrP^C^ synthesis in the cell.^35^^,^^36^^,^^37^^,^^38^^,^^39^ Due to the UPR activation relying on the endoplasmic reticulum signaling pathways, we assumed that replication and accumulation of prions along the axons do not contribute to the UPR mechanism. We therefore defined a zone surrounding the soma of the neuron where accumulation of misfolded proteins transiently induces UPR activation^71^ (Figures 1C and 1D).

Like most cell regulation systems, the UPR is bistable.^54^^,^^71^^,^^72^ In our model, it is triggered when the number of misfolded assemblies ($A+\sum_{i}^{n}B_{i}$) in the UPR control zone exceeds a threshold (denoted σ).^54^ While the UPR of a neuron is activated, no templating reactions can occur in its associated templating field. The resilience (re-activation of templating field) occurs when the total number of misfolded assemblies in the control zone remains under the threshold for a delay τ (Figure 1E). Both σ and τ are parameters specific to a given cell-type and could characterize a given brain area.

#### Gillespie stochastic reaction-diffusion modeling

Based on the findings previously mentioned, modeling prion dissemination in brain tissue requires considering autocatalytic dynamics, replication, and tissue response, which vary with position relative to neurons. These elements characterize prion dissemination as a reaction-diffusion system, governed by the combination of local chemical reactions and extracellular diffusion of the assemblies.

To model such a system, a suitable approach involves adapting the Gillespie algorithm, a stochastic simulation method that predicts the behavior of chemically reacting systems by randomly determining the timing and outcome of each reaction event.^73^ Although it wasn’t originally designed to include diffusion, it can be modified by dividing the space into compartments, in our case, a $N_{vox}\times N_{vox}$ square grid of voxels. Each compartment has state variables for the number of reactant molecules it contains, allowing for the calculation of all possible events, including reactions within compartments and diffusion between neighboring compartments. Random numbers are then used to select the next event and its time of occurrence based on their probability distribution. The system is updated based on the selected event, and time is incremented. Repeating this process simulates the combined effects of reactions and diffusion over time.

Given $X_{c}={\left[A\;B_{1}\cdots B_{n}\right]}_{c}$ the state variable of compartment *c*, i.e., the number of each reactant molecule it contains (note that this approach requires to introduce a maximum *B*_*i*_ assembly size *n*, we kept *n=10* in all simulations), the propensity of the reaction $A+B_{1}\to 2B_{1}$ occurring in compartment *c* is $\alpha =\lambda A_{c}B_{1c}$, where λ is the reaction rate constant. Additionally, given $D_{A}$ the diffusivity of *A* assemblies, the propensity of the event corresponding to the diffusion of *A* outside of compartment *c* is $\alpha =\frac{4D_{A}}{L^{2}}A_{c}$, where *L* is the length of the square-shaped voxels in the 2D domain. As diffusion is considered to be isotropic in this model, an element exiting a compartment has an equal chance to jump to any of its 4 neighbors, as the domain is divided into square-shaped voxels. Finally, given $\alpha_{0}=\sum \alpha_{j}$ the sum of the propensities of all events across all compartments (reaction and diffusion), the probability of event *j* of propensity $\alpha_{j}$ occurring is $p_{j}=\alpha_{j}/\alpha_{0}$ and the time until the next event τ is exponentially distributed, with exponential rate being $\alpha_{0}$. Thus, the simulating method is to draw two pseudorandom numbers, $r_{1}$ and $r_{2}$ on [0,1], and determine the next event *j* and the time until it happens τ using the following equations:

$$\tau =\frac{1}{\alpha_{0}}\log\left(\frac{1}{r_{1}}\right)$$

$$j=\text{the}\;\text{smallest}\;\text{integer}\;\text{satisfying}\;\sum_{j^{\prime }=1}^{j}p_{j^{\prime }}>r_{2}$$

To achieve an accurate simulation, the original application of the Gillespie algorithm requires the reaction environment to be well-mixed so that every reactant can interact with each other. Using discretization of space into compartments, this requirement applies to every voxel individually. To ensure its validity, diffusion events need to greatly outnumber reaction events. This can be controlled using the size of the compartments L which directly impacts the propensity of diffusion events, increasing their likeliness as L decreases.

Changes in assembly size due to templating or intrinsic strain kinetics alter the diffusion coefficients of the assemblies. Here, diffusivity is inversely proportional to the size of the object:

$$D_{A}=D_{0}$$

$$D_{B_{i}}=\frac{D_{0}}{i},i\in ⟦1,n⟧$$

In every simulation, $D_{0}=1000$. Assemblies can exit the system by diffusing outside the domain through one of the edges. This is the only way in the model for assemblies to be eliminated and accounts for the spread of elements to other regions as well as clearance of more diffusive objects *in vivo*.

One of the particularities of this system is that templating reactions can only happen in compartments sufficiently close to neurons, inside their templating field, depending on their UPR status. While the UPR is deactivated, the substrate (PrP^C^) is considered to be in excess and templating reactions for both *A* and *B*_*i*_ become pseudo-first orders. Once the total number of assemblies in the UPR control zone exceeds the threshold σ, i.e.,:

$$\sum_{c\in UPR\;zone}\left(A_{c}+\sum_{i=1}^{n}B_{ic}\right)>\sigma$$

The UPR activates and replication is then shut off in the compartments inside of the templating field associated to the neuron. For the UPR to deactivate, the total number of assemblies in the UPR control zone needs to remain under the threshold for a delay τ. While this particular modeling of tissue response using an inactivation delay causes the system to not be a continuous-time Markov jump process, it can still be simulated in a similar fashion by updating the system not only after each event but also whenever a neuron changes UPR state.

#### Simulation parameters

Recent advances in structural biology have revealed distinct atomic-scale structural differences between PrP^Sc^ fibrils from various prion strains.^13^^,^^14^ Consequently, it is anticipated that PrP^Sc^ assemblies from different prion strains will exhibit varied physicochemical properties. In our simulations, we define a prion strain by the set of kinetic parameters that govern the intrinsic dynamics of the assemblies and their replication rates, according to the previously established kinetic scheme.^15^^,^^16^^,^^33^^,^^34^ This kinetic scheme (Figure 1A) is characterized by six kinetic parameters, collectively defining a strain.

Due to the complexity of this highly non-linear kinetic scheme, experimental determination of reaction rates is exceedingly challenging. The parameter values used in our simulations were determined through trial and error, selecting for representative behaviors while ensuring consistency among parameters within a plausible range. Experimental observations further constrained our parameter choices. For example, biochemical assays indicated that subpopulation *A* has a higher replication rate than subpopulation *B*_*i*_,^33^ imposing restrictions on the templating rates. In most simulations, the replication rates were kept constant, except in the co-propagation section, where variability was introduced to assess differences in adaptability to the substrate. The kinetic parameters defining the modeled strains used in this study are summarized in Figure 1B.

The evolution of these modeled prion strains was evaluated on different brain tissue representations using MATLAB. To simulate a brain area, we used a uniform square grid of neurons sharing the same UPR parameters (Figure 1C). The tissue parameters defining a modeled brain area selected in this work were the number of neurons in the N×N square grid and their UPR threshold σ. The UPR deactivation delay τ was kept constant throughout brain area simulations. The parameters defining the modeled brain areas used in this study are summarized in Figure 1F. To mimic the dissemination between two brain areas, we had two clusters of neurons unilaterally connected by axons (Figures 1D and 1G). The tissue parameters studied here were the direction (anterograde or retrograde) and the number of axons linking the two groups.

All our simulations were seeded with the same quantity and distribution of PrP^Sc^ except for the exploration of initial seed amount where the amount had been increased 100-fold. In brain area simulations, the seed was injected in the center of the neuron grid whereas in dissemination experiments through the neural network, it started at either efferent or afferent groups of neurons.
