# Supplementary material for: The dynamics of prion spreading is governed by the interplay between the non-linearities of tissue response and replication kinetics
Source: iScience. 2024 Nov 13;27(12):111381. doi: 10.1016/j.isci.2024.111381 (PMC11664133; doi:10.1016/j.isci.2024.111381)

iScience, Volume *27*

Supplemental information

The dynamics of prion spreading is governed by the interplay between the non-linearities of tissue response and replication kinetics

Basile Fornara, Angélique Igel, Vincent Béringue, Davy Martin, Pierre Sibille, Laurent Pujo-Menjouet, and Human Rezaei

Figure S1. Percentage of replicates maintaining their *A* subassemblies across 50 independent replicates over time for each strain-tissue combination. Related to Figures 2 and 4. Graph (a) corresponds to the nominal initial conditions and graph (b) the increased seeding. Both graphs highlight the transient replication state observed with the evolutions of *S1T2* and *S2T1*, as replicates progressively eliminate subpopulation *A* as simulation time increases. The only notable difference between the two graphs is in the evolution of *S1T1*, where the high initial conditions caused most replicates to eliminate their *A* subassemblies early in the simulations. This shows that initial conditions can impact the evolution of certain strain-tissue combinations.

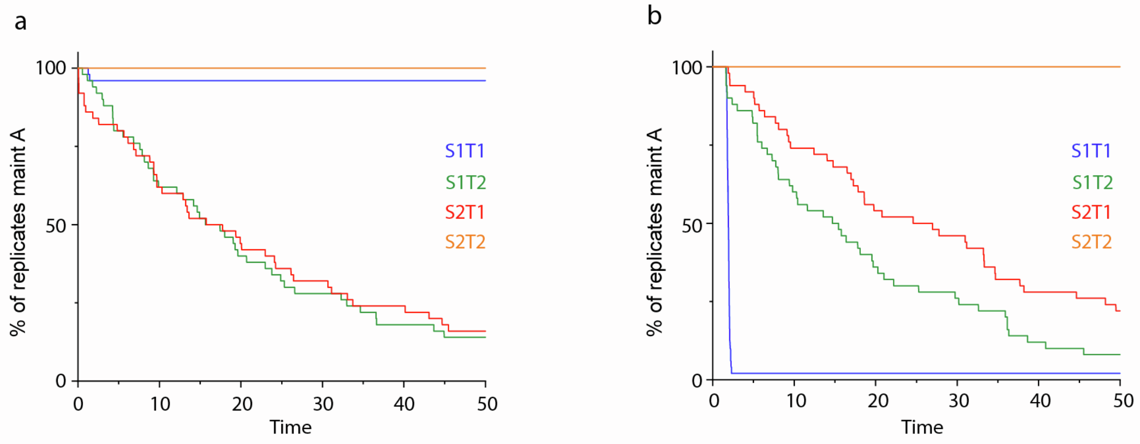

Supplement: Document S1. Figure S1 [file mmc1.pdf]
